# Artificial Intelligence in Brain Tumour Surgery—An Emerging Paradigm

**DOI:** 10.3390/cancers13195010

**Published:** 2021-10-07

**Authors:** Simon Williams, Hugo Layard Horsfall, Jonathan P. Funnell, John G. Hanrahan, Danyal Z. Khan, William Muirhead, Danail Stoyanov, Hani J. Marcus

**Affiliations:** 1 Department of Neurosurgery, National Hospital for Neurology and Neurosurgery, London WC1N 3BG, UK; hugo.layardhorsfall@ucl.ac.uk (H.L.H.); jonathan.funnell.13@ucl.ac.uk (J.P.F.); j.hanrahan@ucl.ac.uk (J.G.H.); d.khan@ucl.ac.uk (D.Z.K.); w.muirhead@ucl.ac.uk (W.M.); h.marcus@ucl.ac.uk (H.J.M.); 2 Wellcome/Engineering and Physical Sciences Research Council (EPSRC) Centre for Interventional and Surgical Sciences (WEISS), London W1W 7TY, UK; danail.stoyanov@ucl.ac.uk

**Keywords:** artificial intelligence, AI, neurosurgery, brain tumour, machine learning, deep learning, surgery, oncology

## Abstract

**Simple Summary:**

Artificial intelligence (AI) is the branch of computer science that enables machines to learn, reason, and problem solve. In recent decades, AI has been developed with the aim of improving the management of patients with brain tumours. This review article explores the role AI currently plays in managing patients undergoing brain tumour surgery, and explores how AI may impact this field in the future.

**Abstract:**

Artificial intelligence (AI) platforms have the potential to cause a paradigm shift in brain tumour surgery. Brain tumour surgery augmented with AI can result in safer and more effective treatment. In this review article, we explore the current and future role of AI in patients undergoing brain tumour surgery, including aiding diagnosis, optimising the surgical plan, providing support during the operation, and better predicting the prognosis. Finally, we discuss barriers to the successful clinical implementation, the ethical concerns, and we provide our perspective on how the field could be advanced.

## 1. Introduction

Artificial intelligence (AI) is the branch of computer science attempting to equip machines with human-like intelligence, enabling their ability to learn, reason, and problem solve when presented with numerous different forms of data. Neurosurgery has often been at the forefront of innovative and disruptive technologies, which have transformed disease course for acute and chronic disease alike [1]. Complex and intricate neurosurgical procedures make the field of brain tumour surgery an ideal candidate for greater integration of AI.

**Figure 1:**
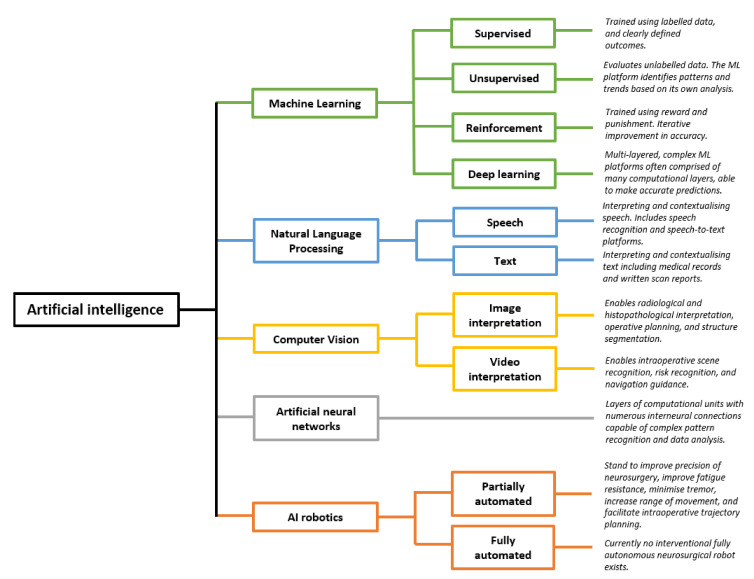
Artificial intelligence and five key subdomains. Each subdomain of AI has numerous potential clinical applications for brain tumour surgery patients. Schematic derived and modified from Panesar et al. [6] and Hashimoto et al. [9]. Numerous other subfields of AI exist, and this schematic is not exhaustive. Please add copyright if necessary.

The term “AI” encompasses numerous components (Figure 1). Machine learning (ML) is the process through which algorithms analyse data and are trained to recognise specific patterns, perform tasks, or predict outcomes. ML may be “supervised”, in which the programmer provides the machine with clearly labelled inputs and outcomes, such that the algorithm may identify trends in predicting these defined outcomes [2]. This requires training data to be labelled prior to its presentation to the algorithms [3,4]. Such an example is by providing an algorithm with numerous features about glioblastoma (GBM) patients, such as age, ethnicity, co-morbidities, stage at diagnosis, and imaging, as well as providing the algorithm with their length of survival employed as an outcome measure. Subsequently, the algorithm will learn and identify the patterns and trends that impact the length of survival, and this can subsequently be used to predict the length of survival in newly presenting patients. This differs from traditional programming, in which a set of rules are ascribed to an algorithm, which then provides an output based on those rules. In ML, only the input and output are provided, while the algorithm “learns” the relevant patterns and trends [3,5]. Alternatively, ML may be “unsupervised”, in which the program analyses data without pre-defined labels, resulting in the ML program identifying similarities between datasets and clustering the data to identify the trends and patterns [2,6,7]. A worked example is the field of radiomics, in which AI programs analyse unlabelled scan images to identify clusters and patterns associated with certain grades of glioma, or by clustering GBM patients who have particularly good outcomes and then identifying the common patterns between these patients [6]. Finally, reinforcement ML is the process through which algorithms are honed based on reward and punishment, whereby actions that increase the likelihood of achieving an end goal are rewarded, and actions distancing the program from the desired goal are punished [6]. The machine subsequently learns the optimal strategy for a given task [8]. With all forms of ML, large volumes of data can be processed leading to the detection of patterns and subtleties indiscernible to clinicians [6].

Numerous algorithms are used in ML, including support vector machines, decision trees, and K-nearest neighbour, though these are beyond the scope of this review [2]. Deep learning algorithms have received particular attention. Deep learning algorithms are multi-layered artificial neural networks made up of numerous computational units that communicate with one another, analogous to a neurons within the human brain, so as to identify highly complex and subtle patterns [9]. 

Natural language processing is the process of enabling machines to understand means of human communication [9]. In a neurosurgical context, examples include algorithms understanding, contextualising, and withdrawing important themes from clinical notation, written reports, and patient histories. This allows large bodies of medical records to be processed rapidly and be incorporated into ML [6,9]. The ability for machines to “understand” human language and subsequently analyse such data is key in integrating human language into predictive models. 

A further branch of AI is computer vision, which can broadly be defined as computer programs interpreting images and videos [9]. Initially confined to image interpretation, the field of computer vision has advanced rapidly through integration with ML neural networks. Novel applications include operative planning and guidance [10,11,12], and real-time “operative workflow analysis” in which steps and phases of procedures are recognised, which may enable the automation of the operative note generation, and provide forewarning regarding high risk stages of the operation [12,13,14]. ML programs capable of image and video analysis have been integrated with surgical robotics, capable of performing tasks with a high degree of precision [15]. This represents a significant paradigm shift, from AI being used as an adjunct to decision making, to AI being used for partially or fully automated interventional procedures [6]. 

AI has the potential to significantly impact the management of brain tumours, with ML, natural language processing, computer vision, and robotics subfields all contributing to novel AI applications that may advance neurosurgical practice. In this review, we present the current advances and potential clinical applications of AI in the pre-operative, intra-operative, and post-operative phases of brain tumour surgery, with a particular focus on intrinsic brain tumours. We also examine specific barriers to further development, as well as current problems with AI in this field. Finally, we consider the medicolegal and ethical standpoints regarding the greater integration of AI in the field of brain tumour surgery.

## 2. Pre-Operative Phase

**Figure 2:**
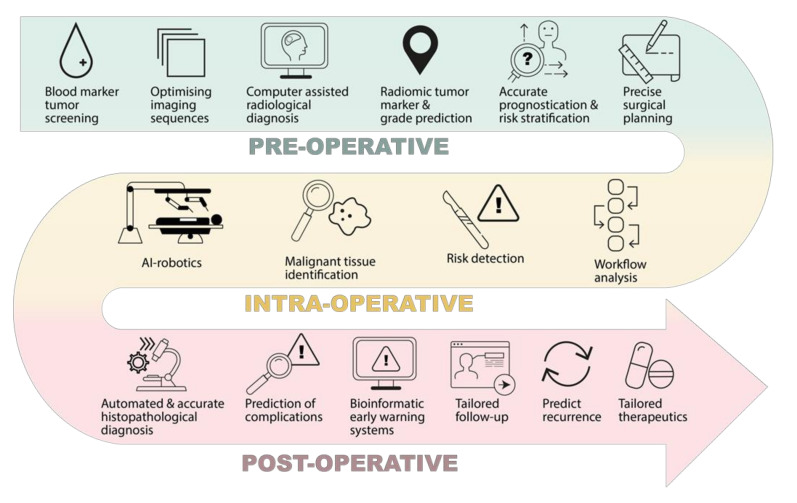
Potential clinical impacts of AI in the neurosurgical management of brain tumours, in the pre-operative, intra-operative, and post-operative phase.

The impact of AI on brain tumour patient outcomes is likely to occur far before the patient reaches the operating table (Figure 2). Research has shown how AI can positively impact various pre-operative stages, such as diagnosis, assessment, and planning [3,6,8,9]. 

### 2.1. Screening and Diagnosis

Intracranial tumours may present with a variety of different symptoms, and at differing stages of disease. Some become symptomatic very early in their development, while others exert relatively little clinical effect despite their growth [16]. The heterogenous degree of presentation may, in part, explain why 30% of brain tumour patients in the UK experience delays in diagnosis [17,18]. Advancements in AI may improve early detection. Developments in recent years have led to the inception of an ML algorithm able to assimilate blood test results to predict and diagnose the presence of brain tumours [19]. The term “brain tumour” encompasses many individual pathologies, yet all exhibit a specific fingerprint on routine blood tests owing to the secretion of various tumour-specific molecules within the neoplastic microenvironment that pervade the blood−brain barrier and enter the wider circulation [20,21,22]. These changes in routine blood tests are subtle, and therefore an ideal candidate for ML analysis. Indeed, ML models have been noted to outperform clinicians in the diagnosis haematological disorders [23]. Podnar et al. used an ML algorithm to detect subtle changes in routine blood tests to detect the presence of brain tumours at the time of symptomatic presentation [19]. Elevated neutrophil count, serum glucose, and decreased eosinophil and basophil counts were among the more significant trends identified by the ML algorithm in the tumour group [19]. Their model showed a sensitivity of 96% and a specificity of 74%, data akin to sensitivity rates for CT and MRI neuroimaging [19,24]. The model was not able to accurately distinguish between primary brain tumours, but was hypothesised by the authors to be a potential screening tool for intracranial malignancy [19]. More recently, Tsvetkov et al. published data regarding an AI-powered detection tool, which uses differential scanning fluorometry of blood samples to detect glioblastoma in patients with a 92% accuracy [25]. As such technology advances, blood markers may feasibly be used to risk stratify brain tumour patients and guide management. This may prove invaluable, in a field in which serum biomarkers to detect and monitor brain tumours have been elusive due to high intratumoural heterogeneity [26]. 

The gold standard of brain tumour diagnosis remains neuroimaging such as MRI. The impact of AI may occur even before radiological images are generated—Brown et al. developed a natural language processing ML algorithm that interpreted MRI brain requests, and subsequently chose the most appropriate MRI brain imaging sequence to generate the most clinically useful images [27]. The ML algorithms significantly outperformed the radiologist sequence choice [27]. Radiological sequences are typically selected by a radiologist, yet protocoling workflow is liable to errors [27,28]. Furthermore, sequence queries for radiographers during working days have been shown to impact radiologists’ time [29] and cause interruptions in image interpretation [30]. ML-based sequence-determining algorithms may play a role in standardising the MRI sequence protocol, thereby maximising the clinical applicability of the scans generated [27,31]. Furthermore, researchers noted that radiologist sequence-selection often struggles with rare conditions, noting that the ML approach performed particularly well in these cases, such as in glioblastoma multiforme [27]. 

Increasingly, radiological images are being recognised as three-dimensional data-sets amenable to algorithmic analysis, deeper than the images seen by individual clinicians [32]. So-called “radiomics” are able to compute swathes of image-based data, down to individual three-dimensional pixels known as “voxels”, to detect and identify patterns that are typically subtle [33]. Thus far, radiomics has been shown to be effective in detecting and characterising a wide range of tumours throughout the human body [32,34,35,36,37]. Specific to brain tumour diagnosis, ML algorithms have been used to characterise the molecular expression of brain tumours [33,38,39,40,41,42], aid in the detection of central nervous system (CNS) metastases [43,44,45,46,47,48,49,50,51,52,53], discriminate between primary and metastatic CNS lesions [54], predict the brain tumour grade [55,56,57,58,59,60,61,62], and predict the presence of genetic mutations [38,63,64,65,66] among other applications [67]. These findings have been demonstrated in a range of CNS tumours, including meningiomas, glioblastoma, and CNS metastases. Further, ML programs have been shown to be superior to the human ability to detect and diagnose brain tumours [67]. 

Specific to intracranial tumours, ML-based imaging interpretation has made great leaps forward in the past decade. The molecular characteristics of gliomas, such as the presence of IDH mutation or 1p/19q mutations, are closely linked to the natural history of the disease, and are known to impact the efficacy of various treatments [68]. The ability to predict these features from imaging data, potentially circumventing the need for invasive biopsies, has been a focus of ML radiomic research for the past decade. Work published by Yu et al. developed an ML algorithm that could predict IDH1 expression status of low-grade gliomas using MRI brain sequences to a high degree of accuracy within 18 min [69]. Molecular diagnosis prior to invasive testing may guide management and predict outcomes for patients, as has been seen to be the case with IDH1 positive gliomas [40]. Chromosomal codeletion of 1p/19q, MGMT promotor methylation status, and IDH1 mutation were collectively detected through a convolutional neural network run by Chang et al. [65]. Many other publications have demonstrated the ability of AI-based radiomic programs to predict tumour marker expression and genetic mutation [39,41,70]. These advances may enable neurosurgeons to offer tailored treatments based on predicted mutations [71].

In recent years, there has been a marked transition away from conventional ML programs to deep learning programs, which integrate numerous layers of neural networks akin to human cortical processing, resulting in powerful systems capable of more complex and subtle pattern recognition [72]. Comparisons in the accuracy of brain metastases detection showed that the deep learning group had a statistically significantly lower rate of false-positives per person when compared with conventional ML, suggesting an ever growing accuracy [72].

### 2.2. Planning

Prognosticating and risk stratifying is a vital component of managing patients with brain tumours. Exposing patients with advanced disease to invasive interventions that do little to prolong their survival or quality of life is deleterious to patient wellbeing. As such, due consideration should be given for deciding in which patients surgery is appropriate. Predicting survival for patients with CNS tumours is difficult, yet it is often the most important question for patients and relatives [73]. At present, numerous scoring systems such as performance status exist to predict survival, yet these often fail to individualise their predictions [74]. ML has been shown to accurately predict survival for CNS tumour patients in a range of settings [39,70,75,76,77,78,79,80,81,82]. Oermann et al. used an artificial neural network to integrate data including patient age, the presence of systemic disease, primary tumour type, and number of metastases to predict the one-year survival in patients with brain metastases treated with radiosurgery. Their model outperformed traditional means of predicting survival [74]. In 2021, Ko et al. demonstrated the ability of an ML platform to accurately predict the progression and recurrence of meningiomas from radiological data alone [81]. Evidently, AI multivariate programs have the potential to individualise predictions, thus supporting the delivery of patient-centred care [73]. 

Key advances have also been made in radiomics with regards to operative planning. Surgical management, particularly in high grade glioma, remains controversial, and the decision as to whether to biopsy versus resect is not guided by high quality evidence. Invariably, cases are judged on an individual basis, balancing the benefits of resection with the risk of neurological impairment, as is seen in approximately 10% of GBM resections [83]. The risks must not outweigh the benefits, and AI may help delineate this fine balance. Lack of consensus in this field has been demonstrated by Orringer et al., who reported that two neurosurgeons were more likely to disagree with one another than to agree as to whether a GBM would be resectable [84]. Judging brain tumour resectability is challenging. High grade gliomas infiltrate beyond their radiologically evident boundaries, making decisions regarding resection margins high risk [85]. Research has been conducted identifying the five most important anatomical features on T1-weighted MRI sequences in predicting tumour resectability, which has enabled the generation of a validated grading system for predicting tumour resectability [86]. This system has subsequently been integrated into an AI platform, which has been demonstrated to accurately predict the surgical resectability of GBM [87]. In the future, AI platforms such as this may become a vital adjunctive tool to support complex decision-making for surgical selection. 

If a decision is made to operate, a key step is pre-operative trajectory planning. Brain tumours invariably abut surrounding structures, which are vulnerable to damage intraoperatively. These areas may be eloquent, epileptogenic, or at high risk of bleeding; the phrase “the decision is more important than the incision” is never more true than with neurosurgical tumour resection [88]. Traditionally, the identification of these high-risk stages of the operation would be performed through a human interpretation of imaging, as well as intraoperatively. This is time consuming, and research has demonstrated variability between different experts [89]. AI programs have been shown to be effective in accurately segmenting tumours and local structures [90,91]. Notably, in patients with CNS tumours, the cerebral architecture may be significantly distorted, making interpretation by traditional segmentation programs difficult [92]. ML-based algorithms have been shown to be effective in trajectory planning. Work by Dolz et al. investigated the use of deep learning algorithms on MRI to accurately detect local organs at risk for patients undergoing radiosurgery—they found that their automated system was able to accurately segment the brainstem in patients with CNS tumours, and was significantly more time efficient than traditional means [92]. Havaei et al. demonstrated the use of a convolutional neural network that was 30 times faster, as well as being more accurate than state-of-the-art segmentation platforms [93]. Several other publications have also shown success at employing deep learning models to accurately segment cerebral tumours and local at-risk structures [94,95,96,97], as well as intraoperatively model tissue deformation in neurosurgery [98]. Indeed, ML systems have been used to accurately orientate beams in stereotactic radiosurgery [99]. When coupled with radiomic programs, AI could feasibly develop a step-by-step guide for how to approach brain tumours, as demonstrated by the ROBOCAST project [12]. Such trajectory planning has already been shown to be feasible for stereotactic brain biopsies [100]. 

## 3. Intra-Operative Phase

Advancements in AI technology, particularly computer vision, have led to the propensity for ML programs to positively impact brain tumour patients in the intraoperative phase (Figure 2). The main areas of impact include intraoperative tumour identification and workflow analysis. 

### 3.1. Tissue

Intraoperative delineation of the tumour from normal tissue represents a significant challenge to neurosurgeons, and is one with significant consequences. Research has shown that residual peripheral tumour tissue that was not identified and removed intraoperatively is the single most common cause of tumour recurrence [101]. It is unsurprising, therefore, that in glioma and GBM, more extensive tumour resection has been shown to be associated with longer survival [102]. Image guidance has helped to identify tumour location, although accuracy falls during the operation due to the displacement of cortical landmarks [103,104]. Fluorescent tumour markers have been used to intraoperatively discern the tumour from the normal tissue with some success, yet this method is limited to high-grade tumours, and has been shown to result in incomplete tumour resection [105]. Stummer et al. compared the use of the fluorescent marker 5-Aminolevulinic acid (5-ALA) for glioma resection with white light, and noted that with 5-ALA use, 35% of patients have a residual tumour after resection; with white light alone this number rises to 64% [106]. Evidently, even with modern fluorescent techniques, complete tumour resection remains challenging. 

Deep learning platforms integrated with hyperspectral imaging (HSI) offer a solution to the intraoperative identification of brain tumours. HSI combines spectroscopy and intraoperative imaging to provide both spatial and molecular information regarding the surrounding structures [107,108]. The process uses a high-resolution camera directly above the surgical field, which detects visible and near infra-red light to produce hyperspectral digital images [101]. The image pixels then represent microscopic areas of the surgical field. The digital images are then integrated with a deep learning platform, which attempts to delineate the tumour from the normal tissue by detecting microscopic differences in the spectral bands of tissues—the so called “spectral signature”. This technique is non-invasive, and has shown promising results for many different tumour types [109,110,111,112]. Fabelo et al. used this technique on six patients with GBM and employed a deep learning platform comprised of a neural network with three convolutional layers to interpret the HSIs [101]. This method correctly identified the background with 98% accuracy, although the tumour tissue was identified with 42% accuracy [101]. The authors noted that the ability of the technology to binarily classify normal and tumour tissues showed a sensitivity and specificity of 88% and 100%, respectively, suggesting that the technology performed well on correctly classifying the images as being tumour-free [107]. More recently, further research has shown advances in HSI technology [113,114]. Ji et al. demonstrated the use of intraoperative Raman scattering microscopy to identify the tumour from the normal tissue [115]. Their program detects changes in tissue characteristics, such as cellularity, protein:lipid ratio, and axonal density, to assist in detecting neoplastic tissue with a sensitivity and specificity of 97.5% and 98.5%, respectively [115]. Similar AI-based techniques have been demonstrated to provide intraoperative brain tumour histological diagnosis [116].

### 3.2. Workflow

Intraoperative workflow analysis is an exciting area of AI. Such systems use computer vision integrated with ML platforms to track the steps, phases, instruments, gestures, anatomy, and pathology of operations. AI-based workflow analysis has several proposed benefits including intraoperative optimisation of the surgical plan and trajectory, accurate anatomic identification, early warning regarding high risk phases of the operation, standardisation of phases and steps, operative note generation, and contribution to simulation and training programs [117,118,119,120,121]. With the ever-increasing computational power, surgeons may benefit from real-time intraoperative guidance—“avoid this area” and “high risk trajectory”. This technology may, in time, reduce surgical errors, complications, and operating times [117,122]. 

Workflow analysis technology has been demonstrated in the field of brain tumour— after deriving a consensus for the operative steps and phases in pituitary tumour resection [123], Khan et al. demonstrated the ability of a convolutional neural network to detect and analyse operative videos of endoscopic transsphenoidal pituitary adenoma resection videos [124]. The platform was able to detect specific phases with 91% accuracy, and steps with 75% accuracy [124]. As AI continues to develop, it is feasible that intraoperative video analysis and the benefits that this may bring may significantly disrupt existing surgical practise in future [117]. As phase and step recognition platforms become more advanced, natural extensions of AI in this field may develop, such as real-time decision support systems, and partially or fully automated steps of procedures. Surgical robotics integrated with AI have the potential to significantly impact the way brain tumours are managed. While this field remains embryonic, AI-robotics have numerous proposed advantages over existing surgical practise, such as resistance to fatigue, reduction in tremors, and increased precision [6]. In recent decades, a range of neurosurgical robotics have been introduced—the individual analysis of which are beyond the scope of this review [6,15,125,126,127,128,129,130]. Much excitement and promise has been generated around the Da Vinci surgical robot. The Da Vinci robot is the most widely used surgical robot worldwide, and functions as a “master−slave” program in which the operator can remotely control the robot’s many arms to perform minimally invasive procedures [131]. A cadaveric study, however, identified the difficulty in performing minimally invasive cranial microsurgery, citing issues such as poor haptic feedback, limited instrument selection, and cumbersome arms [131]. Pandya et al. showcased the robotic system NeuroArm, a surgical robot capable of image-guided microsurgery, which represents a highly promising AI-robotic platform [15]. At present, there remains a plethora of barriers, such as cost, workflow integration, and additional training [132]. The inception and integration of an autonomous surgical robot capable of human surgical performance for CNS tumours remains unlikely in the near future [6,133].

## 4. Post-Operative Phase

The unique ability of AI programs to assimilate large volumes of data make it well placed to positively impact the post-operative phase, with numerous potential areas of impact (Figure 2). The main areas of impact include inpatient and acute care, and outpatient and oncological care.

### 4.1. Inpatient and Acute Care

The post-operative phase for brain tumour patients is high risk, and is frequently hampered by complications. The development of post-operative complications is dependent upon numerous fixed and dynamic variables, of which ML techniques are uniquely placed to analyse [134].

Numerous examples of AI integration in the post-operative phase have been demonstrated in fields other than brain tumour surgery [135,136,137]. Campillo-Gimenez et al. developed an ML program which used natural language processing to analyse patient medical records, and subsequently develop models for predicting the incidence of surgical site infection (SSI) [138]. Artificial neural networks were used to predict complications such as venous thromboembolism and SSI in patients undergoing anterior lumbar fusion, exhibiting an accuracy of 95%, significantly outperforming traditional logistic regression statistical means (62%) [134]. Hopkins et al. were able to predict the development of SSI in patients undergoing posterior spinal fusion, with a positive predictive value of 92.3% [139]. The authors found that such complications reduce patient satisfaction, incur cost, and worsen outcomes for patients [139,140]. Brain tumour surgery may also benefit from greater AI integration by helping to predict and mitigate the development of numerous other typical post-operative complications, including adverse drug events [141], venous thromboembolism [142], development of pressure ulcers [143,144], falls [145], and hypoglycaemia [146,147]. These complications are all-too-often preventable, and significantly affect patient outcomes. AI has the propensity to reduce the occurrence of these common post-operative issues.

There is also increasing interest in the field of ML in intensive care units (ICU) [148]. Given the large proportion of patients with brain tumours who require admission to ICU in the recovery phase, these advancements may provide support to intensivists by processing the wide range of physiological data present in ICUs. These systems may theoretically detect the deteriorating patient earlier than with traditional methods [148].

### 4.2. Outpatient and Oncological Care

Histological analysis of tumour specimens occurs during the post-operative phase, and is a prerequisite to ongoing oncological care. AI has made significant advances in the field of histology. Traditional approaches to histopathological analysis rely on specimen preparation, staining, assays, and examinations [149]. This process requires human resources and time, which contribute to the delay between tissue sampling and the commencement of rationalised therapeutics. Additionally, existing methods of histopathological diagnosis rely on human visual pattern recognition and analysis of cellular morphological features; despite means of standardisation, this inherently introduces bias due to the subjective nature and differences in judgement between different histopathologists [150,151]. AI stands to disrupt this process, and promises to result in faster, more accurate diagnoses, with more uniform standardization [152]. ML for histological analysis has made significant progress over the last decade [153]. ML programs analyse digitised histopathological slides, and are able to detect both macro and micro patterns, including region texture, shape, and cellular morphology, and process these features to make accurate histopathological conclusions [153,154]. 

AI-based integration into the histological diagnosis of brain tumours has the potential to significantly disrupt traditional pathways. Firstly, AI may alleviate the need for biopsy in the first place, as we have seen the impact of radiomics on predicting the grade and molecular expression as a potential alternative diagnostic modality; secondly, AI has the potential to speed up specimen analysis and to increase the accuracy of grading; thirdly, deep learning models may help us to categorise patients in ways previously unknown to us, which may aid therapeutics and survival; finally, the ability of AI-based programs to predict molecular and cellular markers in tumours may pave the way for highly tailored therapy for brain tumours, thus enhancing the effects of treatment, while reducing unnecessary harm through side effects to patients [150,155,156,157,158,159,160,161,162,163]. An AI-assisted approach to histopathology has been compared against traditional microscopy methods in several studies [150,164,165,166], all of which showed non-inferiority compared with traditional means. With regards to brain tumours, Barker et al. demonstrated the ability of a computer-analysis system to analyse digital histopathology slides and correctly to diagnose GBM and low-grade glioma with accuracy [167]. Ortega et al. used a novel approach of HSI to detect high grade glioma on histopathology slides [168]. Ker et al. used a convolutional neural network to grade brain histology specimens into low grade glioma or high-grade glioma, with 100% and 98% accuracy, respectively [149]. Perhaps most interesting, however, was the use of transfer learning employed in this study. Additional training dataset slides of breast tumours were fed into the AI program, which improved the overall performance of glioma classification. As noted by the authors, this may prove extremely useful when trying to establish ML programs for rare tumours, in which the training dataset is limited [149]. Numerous other studies have shown equally promising results [169,170,171,172,173]. 

The impact of AI in neurosurgical oncology may continue to benefit patients even after discharge during their post-operative recovery. Gvozdanovic et al. developed a ML integrated mobile phone app, Vinehealth, which uses patient inputted data to track symptoms, provide reminders regarding medication and upcoming appointments, and provide tailored educational content [174]. By enabling the patient to regularly input data regarding their own condition, ML-based platforms may gain a far more accurate, real-time understanding of patient wellbeing. In contrast, clinic appointments spaced several months apart often leave room for patient deterioration to go unnoticed. AI-based medication management systems have also been pioneered, aiming to increase adherence [175]. In a similar vein, biometric monitoring systems have become increasingly common in the literature. Such systems use data such as dynamic monitoring of step count and vital signs, allowing for a real-time objective analysis of the patient functional state. These systems have been demonstrated to predict adverse events, hospitalisation, and even changes in depression scores [176,177,178,179,180]. In an era of increasing technological advancement, patients’ phones and Fitbits may soon be vital features of post-operative care. ML programs have also been shown to predict readmission in patients. Such ML algorithms have demonstrated high degrees of accuracy following spinal surgery [181,182,183] and in other settings [184], and may be used in the future to predict which brain tumour patients are at high risk of complication at discharge. 

Adjuvant therapy in the post-operative phase may be fine-tuned by AI programs to achieve maximal efficacy. The choice of modality, dose, timing, and duration of adjuvant therapy has the potential to become highly tailored as AI becomes more integrated with brain tumour therapy. Recently, Yauney et al. described a reinforcement ML program that could iteratively optimise chemotherapeutic dose in a simulated trial of GBM patients [185]. While no explicit evidence regarding brain tumour AI-based chemotherapeutic regimens yet exists, research has emerged in which AI optimises chemotherapeutic regimens at other primary tumour sites [71,186,187,188]. Indeed, the CURATE.AI platform demonstrated the ability of an AI program to optimise the dose and timings of chemotherapeutics in prostate cancer patients, adhering to a narrow therapeutic range [189]. AI models have also been shown to predict the sensitivity of solid organ tumours to chemotherapy [188]. Adjuvant radiotherapy stands to be significantly benefited by more accurate tumour segmentation, as described previously [48,66,89,90,97]. Immunotherapy in CNS tumours remain in the early stages of trials, yet AI platforms may in the future predict response to immunotherapy, as well as optimise the dose and treatment regimen [190]. Furthermore, AI may enable a whole new range of therapeutics to be discovered [188]. ML algorithms can be utilised for high-throughput screening to calculate the probability of a tumour cell line responding to new chemotherapeutics [191]. This reverse engineering of drugs stands to streamline the typically lengthy process of drug discovery, and result in ever-more targeted therapies for brain tumours [188].

Evaluating the response to treatment is key in tailoring therapy for patients. In neuro-oncology, a wide range of parameters are used to monitor the response to treatment, although increasingly, in clinical and experimental practise, response to treatment is assessed using the Response Assessment in Neuro-oncology (RANO) criteria [192]. This criterion relies on post-intervention MRI scanning of brain tumours, and subsequent two-dimensional volumetric analysis of scans. However, such treatment response methods may fail to accurately monitor tumours that exhibit an anisotropic growth pattern, and as such, research has proposed the use of artificial neural networks that monitor volumetric response to treatment [193]. Kickingereder et al. demonstrated the feasibility of such a program and noted superiority in reliability and performance when compared to existing RANO-based methods of response assessment [193]. In the future, therefore, AI may more accurately track response to treatment in brain tumour patients.

## 5. Barriers, Evaluation, and Ethics

While AI has the potential to be transformative in the management of brain tumours, several barriers to widespread introduction exist (Table 1). Furthermore, as the field of AI in neurosurgery expands, a key focus will be the evaluation of novel programs. Evaluation must focus on patient and clinician acceptability, clinical efficacy, and ethical concerns. Barriers to widespread introduction, evaluation of developing AI technologies, and ethical concerns regarding AI in brain tumour neurosurgery are examined below.

### 5.1. Barriers

Firstly, ML models require large volumes of accurate data to be trained. The accuracy and acuity of the data is imperative for devising effective algorithms that represent the clinical setting. Even with effective coding, the use of routine administrative or hospital data in research has its limitations. Furthermore, for supervised ML programs, this data may need to be appropriately labelled and analysed, which is time and labour intensive. The analytic accuracy of a supervised ML algorithm is only as good as the data provided, and therefore access to large volumes of accurately labelled data may prove to be a significant barrier in the introduction of AI to neurosurgery. Indeed, poor labelling of data has already been shown to lead to diagnostic errors in the field of AI [194]. The need for large training datasets is particularly true in the field of brain tumours, many of which are extremely rare. If AI is to be successfully integrated into neurosurgical oncology, collaboration between institutions, both nationally and internationally, is essential. This has already been the case in the field of radiology, in which the pace of radiomic advancement has been aided by archives of scans of certain pathologies, such as the Visually Accessible Rembrandt Images (VASARI) database of gliomas, and the cancer imaging archive (TCIA) [195]. Through collaboration, video databases of complex operations could be used to train intraoperative risk detection algorithms and aid in the training of robotics [196]. Fundamentally, high quality data must inform algorithms so as to ensure the data represents the problem being addressed. This will require appropriate design, maintenance, and training for managing data. A self-serving solution could indeed be provided by automated data collection systems driven by AI.

A novel solution to the problem of large amounts of training data being required lies in the generation of synthetic image generation to train deep learning models [197,198]. In essence, sufficiently large databases of rare pathology are, inherently, difficult to build, and therefore the generation of synthetic MRI images to mimic their pathology may hasten this process. Shin et al. used a generative adversarial network to augment existing MRI scans, resulting in the generation of synthetic MRI scans demonstrating a specific pathology [197]. This process results in a non-costly diverse dataset, and mitigates concerns regarding the security of patient data that the generation of large, multi-centre repositories results in [197]. However, we must be cautious with regards to use of synthetic data to train AI models, such that ground truth datasets remain rooted in an in vivo pathology. IBM’s Watson for Oncology was trained primarily using synthetic data, and resultantly made numerous erroneous recommendations, several of which posed legitimate harm to patients, such as advising the prescription of bevacizumab in a patient at severe risk of bleeding [199]. 

Importantly, wide collaboration both nationally and internationally would be necessary to generate databases applicable to this diverse group of patients. If algorithms were trained using data from just several institutions confined to one area, the ML program may develop inherent biases [6]. Ever larger databases would help to reduce “framing errors”, in which algorithms are met with situations that are fundamentally different to the dataset with which they are been trained, and subsequently misinterpret the data. The generation of large datasets for training ML algorithms, however, raises concerns regarding patient data security, which would need to be met with rigid safeguards [8].

Collaboration between scientific disciplines should also be a focus for driving the progress of AI in brain tumour surgery. Hashimoto et al. discuss how surgeons should openly collaborate with computer scientists and engineers to steer the development of AI in ways that are both feasible and clinically applicable [9]. Along this line, several publications have drawn upon the importance of sharing promising ML models between institutions [2]. Through open-source coding, promising models may be validated and tested earlier, allowing for bugs and defects to be detected sooner [2]. 

**Figure 3:**
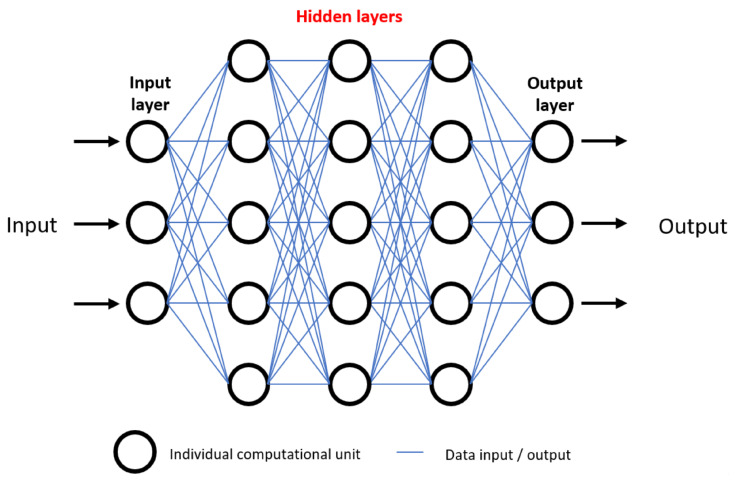
Visual representation of an artificial neural network, demonstrating the “black box” ethical problem. Numerous data inputs are processed among many hidden layers of computational units, ultimately resulting in an output. For example, data inputs may be tumour grade, location, and patient demographics. After data processing, outputs may be survival prediction or response to certain therapeutics. Inability to understand how outputs are generated due to complexity of hidden layers is referred to as the black box problem, and raises concerns regarding trust in deep learning predictive models.

As ML algorithms and neural networks become more advanced, their decision making and predictive abilities become more difficult to unpick. As mentioned previously, neural networks consist of a varying number of layers of computational units. Data are inputted, followed by a series of “hidden layers” in which neurons are able to interact with one another, followed by an output (Figure 3). The “black box” conundrum refers to the fact that, through design, AI neural networks detect patterns and interactions in a wide range of variables undetectable to humans, and therefore humans may be left with an inability to evaluate how and why a conclusion was reached [9]. Without a proper understanding of how predictions are made, the following question has been posed: “why should I trust you?” [200]. This is a legitimate concern, as clinicians may find themselves blindly trusting an algorithm, with disastrous consequences. As we have seen, despite the vast potential for AI to improve outcomes in patients, it is not without faults, and as such there should be a drive to improve interpretability of AI algorithms in future, enabling us to peer inside the “black box” [8].

Understanding specific neural networks will be crucial as AI becomes more widespread in the management of brain tumours. As Panesar et al. note, at present, surgical errors involve one patient at a time; with a greater integration of AI into systematic decision making and prediction systems, the potential for systematic errors and mistakes to be made on a population wide level is concerning [6]. This has occurred in practise, when IBM Watson Health’s algorithm for cancer management advised erroneous treatment strategies for patients [199]. 

Finally, the inability of AI to deal with uncertainty remains an issue. Cabitza et al. explain that ML-based systems use categorical or numerical data as their input and cannot process the notion of uncertainty. It is common for two clinicians to disagree with one another regarding the diagnosis or management strategy for a patient, and this is not necessarily due to clinician error, but instead due to the intrinsic uncertain nature of modern neurosurgery [201]. This clinical uncertainty can guide management plans (perhaps a clinician takes a cautious treatment approach or refers the patient for a repeat scan) in ways that AI platforms cannot. 

Further to these issues, the implementation of AI in neurosurgery faces other more immediate practical challenges, such as the technological infrastructure required to accommodate such technology, and financing the introduction of AI technology in healthcare. As discussed, AI is an umbrella term that encompasses numerous different programs with numerous different applications. Each practical application of AI may require differing infrastructure needs. For example, radiomic analysis of brain tumour MRI sequences may require the installation of software capable of performing this analysis. This may seem like an achievable step, however ensuring that healthcare providers have the hardware to operate these algorithms is another issue, and one which may be highly costly. Indeed, such is the computational power required to run many AI platforms, and widespread use may require an overhaul of healthcare IT systems. Moreover, the development of an AI-enhanced, semi-automated robotic operating device clearly represents several challenging steps in terms of both hardware and software infrastructure development. 

A greater integration of AI will evidently require large injections of both human capital and funding. The source of this funding remains another potential barrier. Costs must be carefully balanced against the projected gains of AI implementation. While reports have suggested that AI stands to result in significant economic savings [202], there have been no economic studies regarding AI in brain tumour surgery. It is imperative that future AI applications are regularly assessed for their cost-effectiveness.

The widespread introduction of AI in brain tumour surgery will require the addition of AI to the neurosurgical and oncological teaching curriculum. At present, neurosurgical teaching curricula has little focus on AI as a teaching subject, with research demonstrating that one third have no prior knowledge of the subject [203]. As clinicians, we must be expected to understand the mechanisms of the technology we operate, such that black-box systems are not perpetuated. Therefore, education of the next neurosurgical generation in the applications of AI must be seen as a key goal toward implementation. 

Despite their promise, the role of AI in brain tumour surgery evidently faces many challenges. Critical evaluation of the developing technologies needs to be thorough to ensure that AI remains a transformative force for good in neurosurgery.

### 5.2. Evaluation

All developing AI technologies should be fully understood, such that their outcomes and predictions can be traced and understood, thus avoiding the “black box” conundrum (Figure 3). Subsequently, all new technologies should be rigorously reviewed both mechanistically and ethically. Existing approval procedures for medical devices (such as FDA approval) are often slow, and fail to appreciate the nuanced risk−benefit considerations when dealing with a disruptive technology [204,205]. As such, we advocate for frameworks such as IDEAL to be employed by researchers, which enable a graduated degree of integration of innovative devices or technologies [206]. Such frameworks are necessary to promote the development of innovative surgical devices, as research has shown that just 9.8% of novel surgical devices make it to a first-in-human study by 10 years [207]. 

The DECIDE-AI Steering Group have called for close evaluation of AI-based platforms as they journey towards clinical applicability. Importantly, they highlight how humans use and interact with AI platforms, stating that ultimately, the most important facet is how clinicians and users follow AI recommendations [208]. They state that the clinical impact of AI technology must be ascertained, before large scale funding of such technology occurs. DECIDE-AI also call for due consideration to be given to new AI platform target populations, rather than just their development populations, and whether significant differences could result between the two [208]. Large-scale clinical trials will help to identify these discrepancies, as well as ascertain the clinical impact of AI, and should be the gold-standard in the future assessment of new AI technologies in the field of neurosurgical malignancy. 

The increasing use of AI in research has prompted the development of AI specific international standards for AI-based clinical trials. The SPIRIT-AI and CONSORT-AI reporting guidelines provide a model to ensure that clinical trials involving AI are both robust and, most importantly, accurately evaluate patient outcomes [209,210]. Furthermore, these guidelines aim to address biases that are specific to AI [209]. 

The ability of AI to process vast amounts of data may not only be applicable to individual cases undergoing surgery for CNS tumours. It is feasible that AI be used to further research and even generate national guidance for certain tumours. In their paper regarding AI in spinal surgery, Rasouli et al. comment on how AI may surpass the current means of guideline generation, which are dependent on the interpretation of large amounts of data combined with clinical expertise by expert panels [127]. Rasouli et al. highlight that the guidance generated is influenced by the quality of data that are presented to the panels, as well as the ability for the panels to accurately pick up on all of the salient points [127,211]. It is easy to see how an analysis of the national archives of data by AI may drive national guideline production in the future.

### 5.3. Ethics

The majority of AI applications thus far have been with complex data analysis, diagnosis, and risk assessment pre-operatively and intra-operatively. Neurosurgical AI-based robotics are an emerging field, although one that is likely to rapidly develop in line with technological advances in the coming decade. With the advent of such pervasive disruptive technologies comes complex ethical questions. 

In the field of robotics, the nomenclature for the degree of robotic autonomy follows the same classification as for the automotive industry, in a six-part scale ranging from level 0, no automation, to level 5, full automation. Level 1 describes some assistance, where AI-based automation is used as an adjunct—a human performs a neurosurgical resection of a cranial tumour, but uses stereotactic systems to help guide the operation. Level 2 describes partial automation, such as the aforementioned “master−slave” surgical robots, in which humans perform the operation, but using surgical robotics. Importantly, a human is still monitoring the procedure throughout and is making decisions. Level 3 describes conditional automation, in which some stages of the neurosurgical procedure are automated, but the procedure is still reliant on the surgeon to perform the remainder of the operation—an example may be the use of an AI platform to position and screw in pedicle screws in spinal surgery. Level 4 describes high automation systems, in which human input is only necessary for troubleshooting or emergencies; the surgical robot is able to evaluate and assess the surgical field and conduct the operation. While a soft-tissue level 4 surgical robot does not exist yet, CyberKnife is an example of this in radiosurgery. Level 5 is full automation, in which a human is not required at any point during the procedure [6,133]. 

Comparisons between autonomous surgical robots and the “driverless car” field do not end at nomenclature, and indeed progress in the two fields is often mirrored. Public opinion has been varied with regards to driverless cars, with concerns raised regarding the issue of liability [212]. Should a semi-autonomous car crash, there is significant debate as to who should be accountable. If humans were to have some control, would they be liable for all the damage? This has been dubbed the “moral crumple zone”, describing humans being disproportionally penalised for complex human−AI interactions over which they have limited control [213]. The “Moral Machine” experiment, published in 2018 by Awad et al., tried to elucidate the public perception of autonomous cars in a worldwide survey in which participants were walked through a series of ethical dilemmas, such as who should autonomous cars prioritise in the event of emergency [214]. The study found considerable variation in ethical standpoints between cultures and geographical location, highlighting the complexity of the discussion [214]. 

Autonomous surgical robotics (level 3 or above) are very much in their infancy, although they are rapidly advancing. Ethical concerns have arisen regarding mistakes, errant robotic behaviour, and poor outcomes. Where does the culpability lie in these instances? Are neurosurgeons and, more importantly, the public, comfortable handing over autonomy to our machine counterparts? Several studies have explored these themes, including the iRobotSurgeon survey [213], among others [215,216,217]. While patients have generally positive attitudes to AI being used in an assistive or diagnostic role, concerns arise when discussing fully autonomous robotics [215]. Fears regarding systems losing control, accurately detecting risk, and humans being replaced by superior technologies are all recurring themes in the literature [215,218,219,220]. Conversely, patients are generally supportive of the use of robotics and AI being used in an assistive role in neurosurgery [215]. This rapid development also leads to questions regarding legal culpability, of which there is little precedent [213]. An intraoperative error by an autonomous or semi-autonomous robot that causes significant harm to a patient is legally a grey area, with culpability feasibly falling upon the surgeon, the software developer, or the hardware manufacturer [216]. Recently, the EU introduced policy regarding AI and robotics, a key tenet of which is algorithmic transparency [221]. 

While the term “artificial intelligence” has increasingly crept into the layperson lexicon in the past decades, research has shown that one quarter of the public are entirely unaware of what AI is, while just over half (55%) are able to give a reasonable definition of the field [215]. Public awareness and perception will be shaped by media portrayal [222], and it is important that accurate portrayals of AI are presented to the public. With such a disruptive technology, headlines touting “killer robots” [223] may lead to resistance to its wider introduction and skew public opinion away from the legitimate benefits that AI poses [222]. Delicate discussions need to be had with the public, engaging their wider concerns, while also presenting the plethora of benefits AI poses. Indeed, numerous studies have commented on the need for the public to build trust with AI robotics [218,224]. Palmisciano et al. [215] cited the following three key recommendations for improved public engagement with AI:

(1) New AI systems must be introduced in gradual phases to the public, with emphasis placed on their role in assisting rather than performing autonomously.
(2) The scientific community should engage in a clear and transparent discussion with the wider public, highlighting the benefits and specific functions of AI.
(3) Statistical data from prior testing should be provided to the public to support the case for the safety of AI [215].

Public opinion may be shaped by media portrayal, but for the most part carries legitimate concerns. These concerns should be used to guide development of AI in neurosurgery, and should ensure that the field continues to question how far can we go versus how far should we go. One such concern is the notion “uniqueness neglect”—the inability of AI to accurately contextualise data and weigh up the current psychosocial status and unique circumstance of patients [225]. This may serve as a reminder to the scientific field of the importance of the doctor−patient relationship, and technological advances that pose a threat to this must be met with significant scrutiny. 

Widespread integration of AI into the management of CNS tumours may result in deskilling of neurosurgeons [6]. While this is certainly unlikely to happen soon, we may see a gradual increase in the proportion of cases performed by robotic or computer-assisted means. Lessons may be learned from fields in which AI has been transformative, such as in aviation. Several incidents have occurred in recent years in which pilots’ over reliance on computer-based flight systems have been highlighted. Panesar et al. note that, paradoxically, while AI has significantly increased safety within the aviation industry, this over-reliance has produced a significant de-skilling of operators, which only becomes apparent in times of emergency and ML failure [6]. Neurosurgeons must endeavour to use ML as an adjunct and continue to maintain their surgical skills to a high degree, to avoid de-skilling. 

Concerns regarding clinician displacement and replacement by AI robotics —the “human-vs.-machine” paradigm—are unlikely to bear truthful in the near future, and indeed, research has identified that neurosurgeons are generally comfortable with greater integration of AI [8,203]. Humans and ML models are likely to work in tandem with one another, rather than be fully replaced. Importantly, the neurosurgical field should ensure that trainees are given adequate training with newly introduced AI, such that they understand it fully in times of emergency. In doing so, neurosurgery can protect from mishaps and learn lessons from the integration of AI in the aviation industry [6]. 

Overall, it is likely that AI will positively impact the field of neurosurgery for brain tumours in the coming years, with clinical applications already being realised. However, we should be cautious of unintended consequences. The litmus test for AI platforms in our field should not only be metrics regarding their accuracy and clinical performance, but should also be clinician and patient satisfaction with the technology [201]. This is because firstly, clinicians must buy-in to the technology to support its adoption and diffusion among the neurosurgical community. Secondly, patients must be willing to consent and engage in treatments supported by AI technology. Regular and stringent analysis of patient and clinician acceptability should be employed to mitigate the risk of unintended consequences.

## 6. Conclusions

AI has the potential to revolutionise the way in which patients with brain tumours are managed. This will be at all phases of the patient pathway: (1) pre-operative screening, diagnosis, and treatment planning; (2) intraoperative tissue analysis and intraoperative workflow analysis; and (3) post-operative acute phase, and outpatient and oncological care. Furthermore, AI may alter the way in which national guidelines are generated, as well as aiding research into brain tumours and therapeutics. In doing so, AI will improve clinical outcomes for patients in years to come. 

Numerous barriers to the development of AI in the field of brain tumour surgery exist. As the field rapidly expands, collaboration will be key in developing clinically applicable AI. Such collaboration should focus on the development of databases and repositories that may be used to train further AI. As ML algorithms become more advanced, open access to such algorithms should be mandatory to encourage wider technological advancement. As AI platforms relating to brain tumour surgery develop, clinical trials should conform to reporting guidelines to ensure robust evidence and reduce biases.

While AI promises to improve patient management, there remain valid concerns regarding the increased integration of machines in modern neurosurgery. Improvements in patient outcomes may be countered by physician deskilling, job replacement, and uniqueness neglect. Stringent patient and clinician acceptability should be sought in the coming years, to ensure that the double-edged sword of AI does not precipitate unintended consequences.

## Figures and Tables

**Table 1:** Barriers and solutions for integration of AI into brain tumour surgery.

Barrier	Proposed solution
Requirement of large datasets to train existing ML programs	Creation of international databases as repositories for training data for brain tumours. Collaboration between neurosurgical oncology units. Synthetic multi-parametric MRI image generation.
Selection bias of training data	Ensure a wide range of demographics used to train ML programs. Use of international databases as repositories for training data.
Patient confidentiality concerns when sharing patient data between units to train ML platforms	Robust scrutiny of data governance for existing databases. Development of technologies in accordance with existing ethical and legal frameworks. Synthetic multi-parametric MRI image generation.
Slow progress in advancing ML programming	International collaboration between ML programming teams. Publishing code for all newly developed ML platforms, making code widely available for further development and scrutiny.
“Black box” conundrum	Ensure that human users can understand and trace all predictions and decisions made by future ML platforms.
Poor contextualisation of uncertainty by ML programs	Ensure that ML platforms developed for use in brain tumour management are used in tandem with clinicians, who are better able to contextualise and explain uncertainty.
